# A Closer Look at the *Choricotyle chrysophryi-like* (Polyopisthocotyla: Diclidophoridae) Species Complex: Description of a New *Choricotyle* from the Gills of *Pagellus acarne* (Teleostei: Sparidae) and Revision of *Choricotyle* spp. from Sparids

**DOI:** 10.1007/s11686-025-00999-z

**Published:** 2025-03-04

**Authors:** Chahinez Bouguerche

**Affiliations:** 1 Department of Zoology, Swedish Museum of Natural History, Box 50007, 104 05 Stockholm, Sweden; 2 Institut Systématique Évolution Biodiversité (ISYEB), Muséum National d’Histoire Naturelle, 43 Rue Cuvier, CP 51, 75231 Paris Cedex 05, France

**Keywords:** Monogenea, Polyopisthocotylea, Sparids, *Choricotyle*, Phylogeny, Atlantic, Mediterranean

## Abstract

**Purpose:**

The genus *Choricotyle*, the largest in the diclidophoridean family, includes *C. chrysophryi*, whose taxonomic status remains ambiguous. This study aims to resolve this ambiguity by describing a new *Choricotyle* species, *C. justinemusei* n. sp. previously identified as *C.* cf. *chrysophryi*, and clarifying the taxonomic status of related congeneric species, particularly those reported from sparids in Mediterranean and Atlantic waters.

**Methods:**

*Choricotyle justinemusei* n. sp. was described based on Mediterranean specimens from the gills of *Pagellus acarne*, found in the Muséum National d'Histoire Naturelle, Paris. The species was differentiated from its congeners through morphological and anatomical features, including the presence or absence of a terminal lappet, and of ring organ, number of atrial hooks and of testes. Molecular analysis using *cox*1 sequences was also conducted to aid in species identification.

**Results:**

*Choricotyle justinemusei* n. sp. was described and distinguished from other *Choricotyle* species by several key morphological traits and molecular sequences. The record of *C. chrysophryi* from *Pagellus bogaraveo* in Atlantic waters was reassigned to *C. chrysophryi* *sensu* Llewellyn (1941). Furthermore, *C. pagelli* from *P. bogaraveo* was found to be distinct from *C. chrysophryi sensu* Llewellyn (1941), confirming the validity of both *C. pagelli* and *C. chrysophryi* as separate species, and the former was reinstated as a valid species. A differential diagnosis was also provided for *C. marionis*, reinstating it based on its original type-host, *Spicara maena*.

**Conclusion:**

This study clarifies the taxonomic status of *C. chrysophryi* and related species, describing *C. justinemusei* n. sp. and reinstating *C. pagelli* and *C. marionis* as valid species. These findings contribute to a more accurate understanding of *Choricotyle* species and their host specificity.

## Introduction

The genus *Choricotyle* Van Beneden and Hesse, 1863, type genus of the subfamily Choricotylinae Sproston, 1946, is the largest in the diclidophoridean family [1]. Among the genera included in this subfamily, *Choricotyle* was considered “the central genus of the subfamily” as it showed to be a composite polyphyletic or, rather, paraphyletic group [1]. To date, 25 valid species have been accepted in this genus [2]. Despite it being “the most primitive representative of choricotylinean” [1], the validity of several species should be verified. In his early effort on the systematics and phylogeny of the family Diclidophoridae Cerfontaine, 1895, Mamaev [1] pointed out that *Choricotyle* warrants a comprehensive analysis and reassessment of its composition. Moreover, the taxonomic composition of the genus remains problematic, with significant disagreement among authors regarding the validity of various species [3]. Many species within this genus have been repeatedly reassigned between *Diclidophora* Krøyer, 183 [4–8] and *Cyclocotyla* Otto, 1821 [7, 9], further complicating its classification.

Overall, *Choricotyle* spp. are known to exist in fish hosts members of the family Haemulidae to a large extent (Table 1). The second most common host group is sparids, in representatives of three independent fish genera: *Pagellus* Valenciennes, *Spicara* Rafinesque, and *Pagrus* Cuvier. However, considering the available data, it's unreasonable to define a host specificity pattern for *Choricotyle* spp. from sparids. Morphometrical data for populations for which sequences are available, along DNA and morphological data from type-hosts and type localities, will certainly lead to a better assessment of host specificity within *Choricotyle* spp.

**Table 1:** Host and localities of *Choricotyl*e spp. (valid species only)

Polyopisthocotyla	Type-host	Habitat in host	Type locality	Source
*C. anisotremi* Oliva, 1987	*Anisotremus scapularis/*Haemulidae	Gills, operculum inner	Chile, SEP	[49]
*C. aspinachorda* Hargis, 1955	*Orthopristis chrysoptera/*Haemulidae	Gills, pharynx parasitic cymothoid	North Carolina, WCA	[50]
*C. australiensis* Roubal, Armitage and Rohde, 1983	*Pagrus auratus*/Sparidae	Gills	New South Wales, SWP	[51]
*C. brasiliensis* Luque, Amato and Takemoto, 1993	*Orthopristis rubra*/Haemulidae	Gills	Brazil, SEA	[52]
*C. caudalis* (Koratha, 1955)	*Leiostomus* xanthurus/Sciaenidae	skin of caudal region	Gulf of Mexico, WCA	[4]
*C. caulolatili* (Meserve, 1938)	*Caulolatilus princeps*/Malacanthidae	Gills	Galapagos island, SEP	[5]
*C. chrysophryi* Van Beneden and Hesse, 1863	*Sparus aurata*/Sparidae	Gills	Brest, NEA	[10]
*C. elongata* (Goto, 1894)	*Pagrus* tumiformis/Sparidae	Mouth cavity, or on parasitic *Cymothoa*	Japan, NWP	[6]
*C. hysteroncha* (Fujii, 1944)	*Haemulon striatum*/Haemulidae	Gills	Florida, WCA	[7]
*C. isaciencis* Oliva, González, Ruz and Luque, 2009	*Isacia* conceptionis/Haemulidae	Gill filaments	Chile, SEP	[53]
*C. labracis* (Cerfontaine, 1895)	*Dicentrarchus labrax*/Hexagrammidae	Gills	North Sea, NEA	[7]
*C. leonilavazquezae* Lamothe-Argumedo, Aranda-Cruz and Pérez-Ponce de Leon, 1998	*Microlepidotus brevipinnis*/*Haemulidae*	Gills	Mexico, ECP	[3]
*C. marionis* Saint Loup, 1885	*Spicara maena*/Sparidae	Not available	France, WM	[8]
*C. multaetesticulae* (Chauhan, 1945)	*Pellona* sp./Pristigasteridae	Gills	India, WIO	[9]
*C. oregonensis* McCauley and Smoker, 1969	*Antimora rostrata*/Moridae	Gills	Oregon coast ^5^	[54]
*C. orthopristis* Luque, Amato and Takemoto, 1993	*Orthopristis rubra*/Haemulidae	Gills	Brazil, SEA	[52]
*C. pagelli* (Gallien, 1937)	*Pagellus bogaraveo*/Sparidae	Gills	British Isles, NEA	[8]
*Choricotyle pellonae* Kritsky and Bilqees, 1973 ^3^	*Ilisha* elongata/Pristigasteridae	^2^	^2^	[1]
*Choricotyle polynemi* Mamaev, 1972	*Polydactylus sextarius*/Polynemidae	^2^	^2^	[1]
*Choricotyle rohdei* Cohen, Cardenas, Fernandes and Kohn, 2011	*Ctenosciaena* gracilicirrhus/Sciaenidae	Gill lamellae	Brazil, SEA	[55]
*Choricotyle scapularis* Oliva, González, Ruz and Luque, 2009	*Anisotremus scapularis*/Haemulidae	Gill filaments	Chile, SEP	[53]
*Choricotyle simplex* Mamaev, 1976	*Plagiogeneion* microlepis/Emmelichthyidae^2^	^2^	^2^	[1]
*Choricotyle sonorensis* Caballero and Bravo, 1962	*Microlepidotus inornatus*/Haemulidae^1^	^1^	Mexico, ECP	[48]

^1^Original description could not be traced, type-host is provided herein as given in Mendoza-Garfias et al. [48]. ^2^Original description could not be traced, type-host is provided by Mamaev [1]

^3^According to Mamaev [1], *C. pellonae* was called “*C. clupeiphila* sp. nov.” in Mamaev's work of 1972 but since no description was provided, this name should be regarded as *nomen nudum*. No data could be traced for *C. crassicuta* Mamaev and Aleshkina, 1984 and *C. pseudosciaena* Zhang and Xiao in Zhang, Yang and Liu, 2001. *NEA*. Northeast Atlantic, *MWP*. Northwest Pacific, *SEA*. Southeast Atlantic, *SEP*. Southeast Pacific, *SWP*. Southwest Pacific, *WCA*. Western Central Atlantic, *ECP*. Eastern Central Pacific, *WM*. Western Mediterranean, *WIO*. Western Indian Ocean

The type species of the genus *C. chrysophryi* Van Beneden and Hesse, 1863, was first described from the gills of the Gilthead seabream *Sparus aurata* L. from Brest, France, Northeast Atlantic [10]. This species had never been reported on the type-host, yet curiously, frequently reported on other hosts, mainly sparids in Mediterranean waters **(**Table 2**)**. These various hosts records, based only on morphology suggests the possibility of a species complex, as previously demonstrated for the microcotylid *Microcotyle erythrini* Van Beneden and Hesse, 1863 also previously reported from various sparid hosts, other than its type-host the Common pandora *Pagellus erythrinus* L. [11–13]. Another diclidophorid reported on sparids in Atlantic waters is *C. pagelli* (Gallien, 1937), first described as *Diclidophora pagelli* Gallien, 1937 from the gills of the blackspot seabream *P. bogaraveo* (Brünnich) around the British Isles, Northeast Atlantic [8]. Strangely, in all subsequent studies on diclidophorids occurring on sparids including on *Pagellus* or the closely related genus *Pagrus*, there was no mention nor comparison with *C. pagelli,* and all *Choricotyle* from sparids were consistently identified instead as *C. chrysophryi*. Herein, a *Choricotyle* species from the axillary seabream *P. acarne* (Risso), from Mediterranean waters, found in the Muséum national d'Histoire naturelle, Paris (MNHN) differed from its congeners based on morphology and molecules and described herein as a new species. Additionally, we discuss the hosts and distribution of *Choricotyle* spp. and provide upon some nomenclature decisions for *Choricotyle* from sparids.

**Table 2:** Previous host and localities of *Choricotyle chrysophryi* Van Beneden and Hesse, 1863

Host/locality	References
*Sparus aurata*	
Brest, France, Northeast Atlantic	[10]
*Pagellus acarne*	
Montenegro, Central Mediterranean	[32]
Algeria, Western Mediterranean	[33]
Spain, Western Mediterranean	[34]
France, Western Mediterranean	[35]
*Pagellus erythrinus*	
Montenegro, Central Mediterranean	[32]
Turkey, Eastern Mediterranean	[40]
Spain, Western Mediterranean	[34]
*Diplodus vulgaris*	
Montenegro, Central Mediterranean	[32]
*Diplodus annularis*	
Northeast Tunisia, Western Mediterranean	[36]
*Diplodus sargus*	
Montenegro, Central Mediterranean	[32]
Tunisia, Western Mediterranean	[36]
*Oblada melanura*	
Tunisia, Western Mediterranean	[36]
*Spondyliosoma cantharus*	
Turkey, Eastern Mediterranean	[41]
Greece, Eastern Mediterranean	[42]
*Boops boops*	
Turkey, Eastern Mediterranean	[41]
*Pagellus bogaraveo*^1^	
Irish Atlantic Slope and Irish Sea, Northeast Atlantic^1^	[18, 43]
U.K. Northeast Atlantic^1,2^	[44]
Algeria, Western Mediterranean	[29]
Spain, Western Mediterranean	[34]
Algeria, Western Mediterranean	[38]
Algeria, Western Mediterranean	[37]
*Pagrus pagrus*	
Spain, Western Mediterranean	[34]
Algeria, Western Mediterranean	[38]
*Boops boops*	
Tunisia, Western Mediterranean	See [39]

^1^reported as *Pagellus centrodontus*

^2^Referred to as *Cyclocotyla chrysophryi* (Van Beneden and Hesse, 1863)

## Material and Methods

From 2017 to 2019, 45 *P. acarne* were collected from local fishermen in Bouharoun, Algeria, Western Mediterranean (36° 37′ 24ʺ N, 2° 39′ 17ʺ E) as described by Bouguerche et al. (2021) [14]. Fish specimens were transferred to the laboratory shortly after capture and identified using keys [15] and examined fresh on the day of purchase. Gill arches were also resected and placed in separate Petri dishes containing filtered seawater and observed under a dissecting microscope for the presence of polyopisthocotylans [14]. Collected flatworms (17 specimens) were heat-killed, fixed without pressure in near-boiling saline, and preserved immediately in 80% ethanol for parallel morphological and molecular characterization. Nine specimens were processed as hologenophores (*sensu* Pleijel et al. [16]). Hologenophores of *Choricotyle* consist of entire specimens, showing taxonomical features (haptor, testes, and male copulatory organ) and lacking only a lateral part. Whole-mounts for morphological analysis were stained with acetocarmine or paracarmine, dehydrated in a graded ethanol series, cleared in clove oil, and mounted in Canada balsam. The hologenophores (presented as nearly complete specimens with haptor, testes, and male copulatory organ, and missing only a lateral vitelline section) were processed and mounted according to the same methods and deposited at MNHN (see below). Drawings were made through a Nikon Eclipse i80 microscope with DIC (differential interference contrast) and a drawing tube. Drawings were scanned and redrawn on a computer with Adobe Illustrator 2023. Polyopisthocotylans were identified on stained whole mounts [14, 17]. For clamps nomenclature, we followed Llewellyn [18]. For high-level terminology of “Polyopisthocotylea”, we followed the systematics of Brabec et al. [19] who elevated the former subclasses of “Monogenea” to the level of classes, and we use the classe Polyopisthocotyla Brabec, Salomaki, Kolísko, Scholz and Kuchta, 2023.

### Molecular Methods

Total genomic DNA was isolated using the QIAamp DNA Micro Kit (Qiagen). The specific primers JB3 (= COIASmit1) (forward 5′-TTTTTTGGGCATCCTGAGGTTTAT-3′) and JB4.5 (= COI-ASmit2) (reverse 5′-TAAAGAAAGAACATAATGAAAATG-3′) were used to amplify a fragment of 396 bp of the *cox*1 gene [20, 21]. PCR reaction was performed in 20 μl, containing 1 ng of DNA, 1 × CoralLoad PCR buffer, 3 mM MgCl2, 0.25 mM dNTP, 0.15 μM of each primer and 0.5 units of Taq DNA polymerase (Qiagen). Thermocycles consisted of an initial denaturation step at 94 °C for 2 min, followed by 37 cycles of denaturation at 94 °C for 30 s, annealing at 48 °C for 40 s and extension at 72 °C for 50 s. The final extension was conducted at 72 °C for 5 min. The sequence was edited with CodonCode Aligner software version 3.7.1, compared to the GenBank database content with BLAST and were deposited in GenBank as defined by Bouguerche et al. [14].

### Trees and Distances

Phylogenetic analyses were performed using the available *cox*1 sequences of *C.* cf. *chrysophryi* of Bouguerche et al. [14] and those of closely related species available in GenBank **(**Table 3**)**. The Alignment was constructed separately in AliView [22], and trimmed to the shortest sequence. Nucleotide substitution models for phylogenetic analyses using the Maximum likelihood method were estimated using MEGA11 [23]. The Hasegawa-Kishino-Yano model [24] with invariant sites (HKY + I) was used, with 500 bootstraps. The Neighbour-joining (NJ) method [25] was also used for comparison in MEGA11, with 2000 bootstraps. *p*-distances and The Kimura two-parameter distances (K2P) distances [26] were computed from the same datasets with MEGA11. Trees were constructed in MEGA11.

**Table 3:** Collection data for *cox*1 sequences analysed in this study

Polyopisthocotylans	Host	Locality	GenBank	References
*Choricotyle justinemusei* n. sp.	*Pagellus acarne*	Bouharoun, Algeria, WM	MZ127216	Present study^1^
*Choricotyle* cf. *chrysophrii*	*P. acarne*	Sète, France, WM	AY009165	[28]
*Choricotyle chrysophryi*	*P. bogaraveo*	Algiers, Algeria, W WM	OL675213	[29]
*Choricotyle chrysophryi*	*P. acarne*	Bouharoun, Algeria, WM	MZ127222	[14]
*Choricotyle australiensis*	*Chrysophrys auratus*	New Zealand, SWP	MT783685	[56]
*Choricotyle australiensis*	*Chrysophrys auratus*	Australia: New South Wales, SWP	MT783686	[56]
*Choricotyle australiensis*	*Chrysophrys auratus*	Australia: New South Wales, SWP	MT783687	[56]
*Choricotyle anisotremi*	*Anisotremus scapularis*	Coquimbo, Chile, SEP	KJ794206	[57]
*Choricotyle anisotremi*	*Anisotremus scapularis*	Coquimbo, Chile, SEP	KJ794207	[57]
*Cyclocotyla bellones*	*Boops boops*	Bouharoun, Algeria, WM	MZ127224	[14]
*Cyclocotyla bellones*	*Boops boops*	Bouharoun, Algeria, WM	MZ127220	[14]
*Cyclocotyla bellones*	*Boops boops*	Bouharoun, Algeria, WM	MZ127220	[14]
*Allogastrocotyle bivaginalis*	*Trachurus picturatus*	Bouharoun, Algeria, WM	MN192391	[27]
*Allogastrocotyle bivaginalis*	*Trachurus picturatus*	Bouharoun, Algeria, WM	MN192392	[27]

*WM*. Western Mediterranean, *NEA*. Northeast Atlantic, *SEP*. Southeast Pacific, *SWP*. Southwest Pacific

^1^Referred to as *Choricotyle chrysophryi* in Bouguerche et al. [14] and as *C.* cf. *chrysophryi* in Bouguerche et al. [17]

## Results

### Molecular Characterisation

**Fig. 1:**
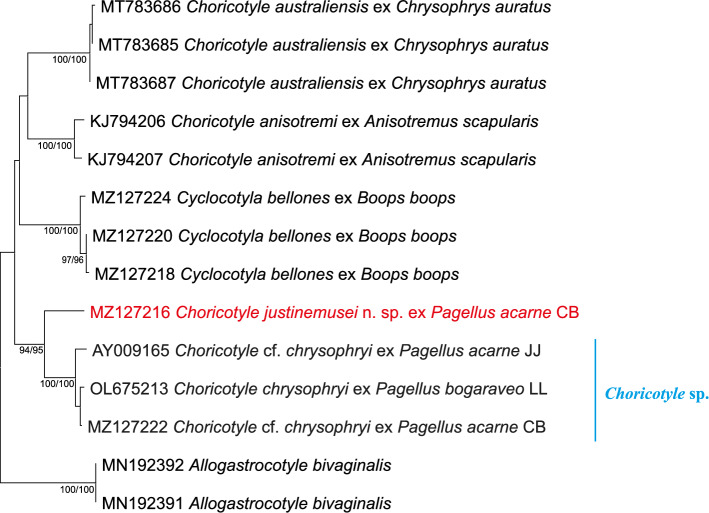
Neighbour-joining phylogram from analysis of the *cox*1 alignment for the Diclidophoridae. Outgroup: *Allogastrocotyle bivaginalis* (Gastrocotylidae). Only nodal support values > 70% are shown. The scale-bar indicates the expected number of substitutions per site. Following Lablack et al. [29], sequence identification and hosts are indicated as in GenBank, followed by a letter: CB, Bouguerche et al. [14]; LL, Lablack et al. [29]; JJ, Jovelin and Justine [28]

A partial *cox*1 (415 pb) sequence of *Choricotyle* ex *P. acarne* from Algeria generated by Bouguerche et al. [14] was aligned with *Choricotyle* sequences available in GenBank and sequences of the closely related diclidophorid *Cyclocotyla bellones* Otto, 1823. In addition to *C. australiensis* Roubal, Armitage and Rohde, 1983 and *C. anisotremi* Oliva, 1987, all available sequences of diclidophorids identified as *C. chrysophryi* in previous studies were included in the analysis. The gastrocotylid *Allogastrocotyle bivaginalis* Nasir and Fuentes Zambrano, 1984 [27] was used as an outgroup. The neighbor-joining and maximum likelihood methods led to similar topologies and thus only the NJ tree is presented in Fig. 1 together with the statistical support from the ML analysis. All the western Mediterranean isolates of *Choricotyle* ex sparid hosts clustered together in a well-supported clade, well separated from *C. australiensis* ex *Chrysophrys auratus* (Forster) from the Pacific (off New Zealand and off Australia), and from *C. anisotremi* ex *Anisotremus scapularis* (Tschudi), also from the Pacific (off Chile).

Sequences of *C*. cf. *chrysophryi* of Jovelin and Justine [28] ex *P. acarne* off France, those of *C*. cf. *chrysophryi* of Bouguerche et al. [17] ex the same host off Algeria, and that of *C. chrysophryi* of Lablack et al*.* [29] ex *P. bogaraveo* off Algeria clustered in a single clade designated herein as *Choricotyle* sp. (indicated in Fig. 1 by the blue line). One sequence of *C. justinemusei* n. sp. (previously designated as *C*. cf. *chrysophrii* by Bouguerche et al. [17]) was highly divergent (see distances below) nested in a sister clade.

Both K2P and *p*-distance were estimated (Table 4). All sequences of *C. chrysophryi sensu lato *, mainly *C*. cf. *chrysophryi* of Jovelin and Justine [28] and that of Bouguerche et al. [17] ex *P. acarne*; and that of *C. chrysophryi* of Lablack et al. [29] ex *P. bogaraveo* differed between them by 1–3% in both K2P and *p*-distances. The sequence of *C. justinemusei* n. sp ex *P. acarne* differed from *Choricotyle* sp. ex sparid hosts by 10–12% in K2P and by 10–11% in *p*-distances. The highest interspecific variation was between *C. justinemusei* n. sp. and *C. australiensis*, ranging between 23–24% in K2P and by 19–12% in *p*-distances.

**Table 4:** Genetic distances between *cox*1 sequences of Polyopisthocotylans

Kimura-2 distances	*C. justinemusei* n. sp.	*C.* cf. *chrysophryi*	*C. australiensis*	*C. anisotremi*
*C.* cf. *chrysophryi*	10–12	*1–3*		
*Choricotyle australiensis*	23–24	20–22	*0–1*	
*Choricotyle anisotremi*	22–23	21–22	16–18	*2*
*p*-distances	*C. justinemusei* n. sp.	*C.* cf. *chrysophryi*	*C. australiensis*	*C. anisotremi*
*C.* cf. *chrysophryi*	10–11	*1–3*		
*Choricotyle australiensis*	19–20	18–19	*0–1*	
*Choricotyle anisotremi*	19	18–19	15–16	*2*

Distances are percentages, and both Kimura-2 and *p*-distances are indicated. Distances within species are in Italics; intraspecific variations are low, ranging between 0 and 3%. The highest intraspecific divergence is between *Choricotyle* cf. *chrysophryi* from *Pagellus acarne* from France (of Jovelin and Justine [28]) and *C. chrysophryi* of (Lablack et al. [29]). These sequences were designated in the tree as *Choricotyle* sp. pending further investigations. Distances between species (interspecific variations) are higher, ranging between 10–12 to 23–24 in K2P and 10–11 to 19–20 in *p*-distances

### Morphology

Class Polyopisthocotyla Brabec, Salomaki, Kolísko, Scholz, Kuchta and 2023

Family Diclidophoridae Cerfontaine, 1895

Subfamily Choricotylinae Sproston, 1946

Genus *Choricotyle* Van Beneden and Hesse, 1863

**Fig. 2:**
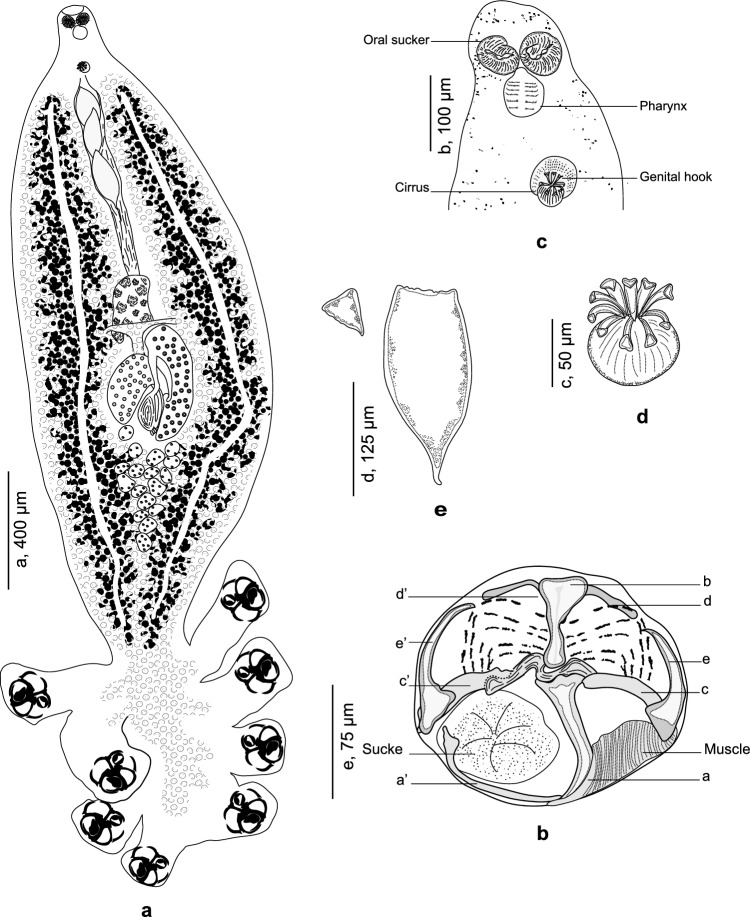
*Choricotyle justinemusei* n. sp. ex *Pagellus acarne*. A, whole body, MNHN HEL1329; B, anterior part showing relative position of prohaptoral suckers and male copulatory organ, MNHN HEL 1329. C, male copulatory organ, MNHN HEL1329; D, egg, MNHN HEL1329; E, clamp, MNHN HEL1333 (habitus redrawn from Bouguerche et al. [17])

*Choricotyle justinemusei* n. sp. (Fig. 2)

*Synonyms*: *Choricotyle* cf. *chrysophryi* of Bouguerche et al. [17].

*Type-host*: *Pagellus acarne* (Risso) (Perciformes: Sparidae), the axillary seabream.

*Type locality*: off Bouharoun, (36 37′24.17″N, 2 39′17.38″E), Algerian coast, Western Mediterranean.

*Site on host*: Gills.

*Type specimens*: Holotype and paratypes are Paragenophores, designated from specimens deposited in the collection of the Muséum National d’Histoire Naturelle, Paris (MNHN HEL1327–HEL1336). Holotype (MNHN HELHEL1329), Paratypes (MNHN HEL1327, MNHN HEL1328, MNHN HEL1328–HEL1336). Specimen corresponding to the sequence (MNHN HEL1310).

*Paratype of specimens with molecular information (hologenophores)*: 1 specimen mounted on a slide, a small lateral part cut off and used for molecular analysis, deposited in the collections of the Muséum National d’Histoire Naturelle, Paris (MNHN HEL1310). The missing part was excised and used for DNA extraction.

*ZooBank registration*: The Life Science Identifier (LSID) of the article is urn:lsid:zoobank.org:pub:6E08CA88-31FA-4E85-8B20-2FA9373DA4C5.

*Etymology*: *justinemusei* is derived from Justine, honoring Jean-Lou Justine, a French parasitologist and former curator at MNHN, and *musei* (Latin genitive form of 'museum'), recognizing the MNHN’s collections and their role in species conservation. This name also acknowledges Justine’s immense contributions as helminths collections curator at the MNHN and commemorates his retirement.

### Description

Based on 14 specimens. Measurements in Table 5. Body stocky, distended in its posterior part and fusiform in its anterior part (Fig. 2a). Anterior end elongated. Haptor semicircular, bearing four pairs of pedunculated clamps. Peduncles short, not containing parts of intestines nor vitelline follicles; length of peduncles decreasing anteroposteriorly, first pair of peduncles the longest. Clamps typically diclidophorid in structure.

**Table 5:** Measurements of species of *Choricotyle* spp. from sparids

Species	*C. justinemusei* n. sp.	*C. pagelli*		*C. chrysophrii sensu* Llewellyn (1941)	*C. australiensis*	*C. elongata*	*C. marionis*
Host	*Pagellus acarne*	*Pagellus bogaraveo*	*Pagrus pagrus*	*Pagellus bogaraveo*	*Pagrus auratus*	*Pagrus tumiformis*	*Spicara maena* *S. smaris*
	Gills	Gills	Gills	Gills	Gills	Mouth-cavity and its parasitic *Cymothoa*	Gills
Locality	Bouharoun, Algeria, WM	Around British Isles, NEA	West Ireland, NEA	Irish Atlantic Slope and Irish Sea, NEA	New South Wales, Australia, SWP	Nagasaki and Hakodaté, Japan, NWP	Bouharoun, Cap Djinet, Réghaïa, Algeria, WM
References	Present study	[8]	[58]	[18]	[51]	[6]	[47]
Body L	4525 (2550–6110)	3000	3400	5000	500–2844 (1381)	8000	4265 (3544–5165)
Body W	945 (825–1125)	1000	1000	1000	24–1264 (810)	1/6 total L	830 (477–1057)
Haptor L	835 (540–1115)	900	900		979–1643 (1106)		1351 (1018–1943)
Lappet L				110			
Lappet W				30			
Oral sucker L	60 (56–70)		350	100 *	53–97 (72)		62 (60–64)
Oral sucker W	50 (42–62)				40–74 (48)		54 (52–56)
Pharynx L	92 (60–126) *				91–123 (102)		94 (92–96)
Pharynx W					72–86 (78)		76 (74–78)
Genital atrium L	39 (25–58) *				40–69 (56)		
Genital atrium W					49–78 (63)		38 (29–53) *
No. of genital hooks	6–10		8		7–9		6
Genital hooks L					16–26 (22)		
No. of testes	25–34	27 from figure					36 (25–47)
Clamps L	233–460				148–220 (188)^a^		245 (235–255)
Clamps W	210–272				123–278 (188)^a^		245 (205–275)

*L.* length, *MCO.* male copulatory organ, *No.* number, *W.* Width, *WM.* western Mediterranean, *NEA.* Northeast Atlantic, *SWP.* Pacific Southwest, *NWP.* Northwest Pacific

*Diameter. ^a^Measurement of largest clamp only provided

Clamps with two regions, an anterior region and a posterior region. Clamps with eight sclerites: *d*, *e*, *c* on the right; *e’*, *c’*, *a’* on the left and two large hollow median sclerites a and b (Fig. 2b). Sclerite *b* I-shaped with two short anterior lobes; sclerite *a* J-shaped curved distally terminating far from the sucker’s margin; sclerites *b* and *a* articulated on each other. Ventrally, sclerite *a* bearing on its distal part a large transversal lamellate extension. Lateral sclerites *c* and *c’* slightly curved, articulated dorsally on proximal part of *a*. Lateral sclerites *d* and *d’* curved, V-shaped, situated in proximal part of the clamp and articulated ventrally to *b*. Median lateral sclerites *e* and *e’* rising dorsally to *c* and *c’*. Proximally, clamps supported dorsally by several small rods. Distally, a well-developed muscle connecting sclerites *e* and *a*.

Mouth subterminal. Oral suckers small, subcircular (Fig. 2c). Pharynx pyriform, immediately posterior to oral suckers. Caeca with lateral branches, extending posteriorly; caeca apparently not confluent posteriorly and do not extend into haptor. Genital atrium mid-ventral, muscular, armed with 9 curved hooks (Fig. 2d) and a globular vesicle. Testes oval, postovarian, limited to the intercaecal space. Vas deferens sinuous, extending anteriorly. Ovary median, complex, and folded. Oötype fusiform. Mehlis’ glands traced in posterior part of oötype. Seminal vesicle voluminous. Oviduct short. Transverse vitelline ducts fused immediately anterior to ovary. Common vitelline duct dorsal to ovary. Vitellarium globular, follicular, co-exiting with intestinal caeca, not extending into haptor. Eggs fusiform (Fig. 2e).

### Remark

**Fig. 3:**
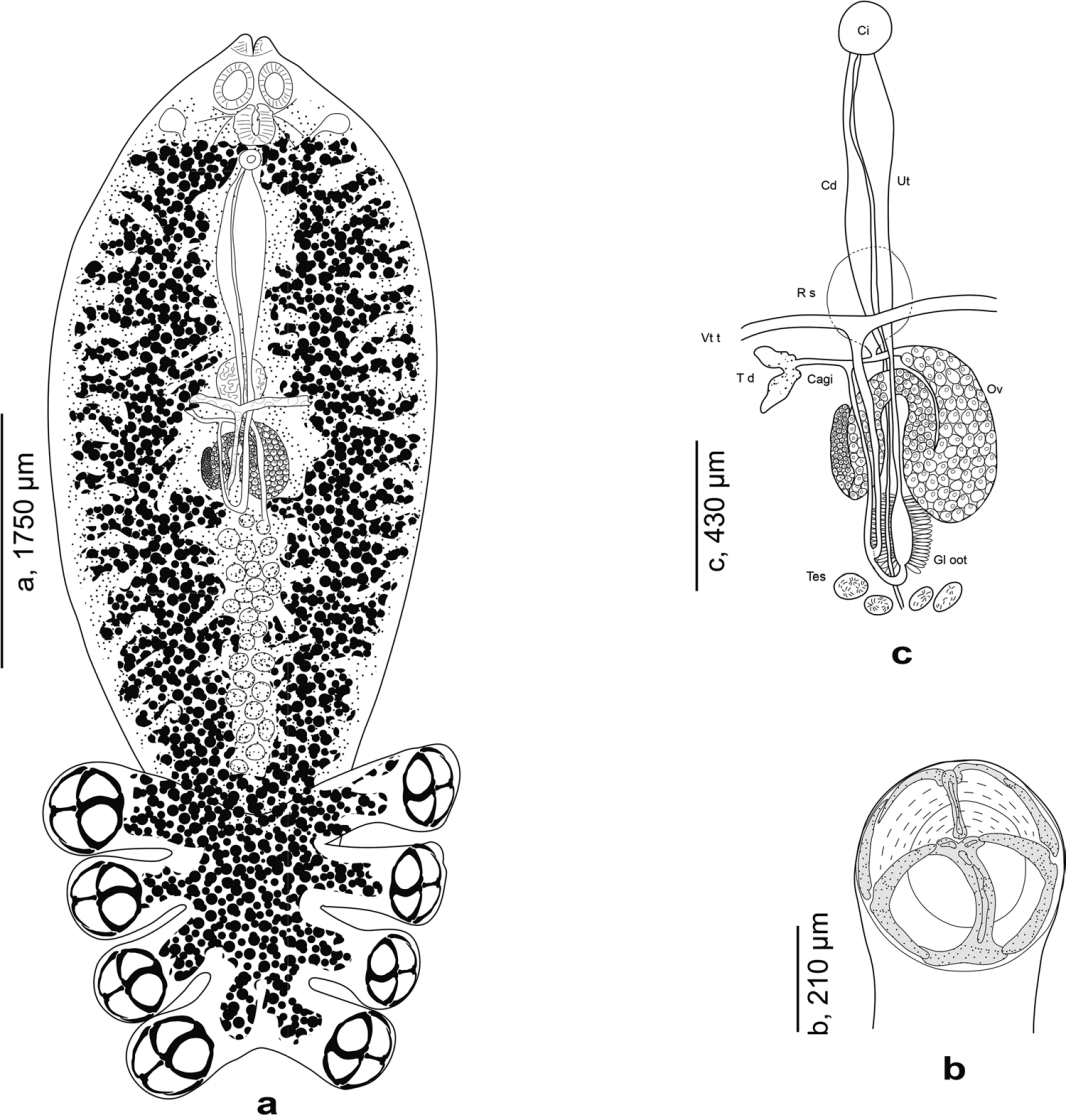
*Choricotyle pagelli* (Gallien, 1937) ex *Pagellus bogaraveo*, reproduced from Gallien [8]. **A** whole body; **B** clamps (referred to as “sucker”); C, Genitals, ventral view

An important record of “*C. chrysophryi*” worth mentioning is that of Llewellyn [18], originally from *P. bogaraveo* off the Irish Atlantic Slope. This record includes detailed illustrations and morphometric data. Notably, Llewellyn [18] emphasized and illustrated the presence of a terminal lappet in his specimens, which is absent in the diclidophorids described by Van Beneden and Hesse [10]. Therefore, the two Atlantic populations are clearly distinct and we refer to the *Choricotyle* from *P. bogaraveo* off the Irish Atlantic Slope as *C. chrysophryi sensu* Llewellyn [18], pending further examination of these specimens. A second species reported in sparids from Atlantic waters is *C. pagelli*, originally described as *D. pagelli* ex *P. bogaraveo* in Northeast Atlantic waters around the British Isles [8] **(see **Fig. 3**)**. It was subsequently found and reported again on its type-host, *P. bogaraveo*, in Portuguese waters of the northeast Atlantic, near the type locality in the British Isles [30], thereby confirming its validity. As a result, we reinstate *C. pagelli* and recognize it as a valid species.

### Differential Diagnosis

*Choricotyle justinemusei* n. sp. differs from *C. pagelli* by having a larger body (2550–6110 *vs*. 3400), smaller oral suckers (56–70 *vs*. 350), less atrial hooks (6 *vs*. 8), more tests (25–34 *vs*. 27^1^), and by the extension of caeca (not extending into haptor *vs*. converging in the haptor). *Choricotyle justinemusei* n. sp. can easily be distinguished from *C. pagelli* by the origins of its anterior peduncles being separated by the width of the body while the origins of its anterior peduncles are contiguous in *C. pagelli.* Moreover, the hosts are different (*P. acarne vs*. *P. bogaraveo*) and the localities are distinct (western Mediterranean vs. Northeast Atlantic). *Choricotyle justinemusei* n. sp. can be easily distinguished from *C. chrysophrii sensu* Llewellyn [18] by the lack of a ring organ at the level of the female genitalia and the lack of the terminal lappet. Similarly, the hosts are different (*P. acarne* vs. *P. bogaraveo*) and the localities are distinct (western Mediterranean *vs*. Northeast Atlantic). *Choricotyle justinemusei* n. sp. is readily distinguished from *C. australiensis* by the number of atrial species (6 *vs*. 7– 9, the length of genital species (10–14 *vs*. 16–26) and the size of clamps (233–460 *vs*. 148–220). Moreover, the divergence in their cox sequences ranged between 23–24 in *p*-distances and 19–20 in K2P. Moreover, the hosts are different *P. acarne* vs. *Pagrus auratus* and the localities are widely separated (western Mediterranean *vs*. Pacific).

## Discussion

The type species of the genus *C. chrysophryi* was first described from the gills of the gilthead seabream *S. aurata* referred to as *Chrysophrys aurata*, in the Northeast Atlantic waters off Brest, France [10]. The fish that Van Beneden and Hesse [10] referred to is certainly not *Chrysophrys auratus* (Forster) (junior synonym of *Pagrus auratus* (Forster) [31]) as they referred to the host also by “*la daurade*”, which is the French vernacular name for *S. aurata*. *Choricotyle chrysophryi* had been frequently reported on other sparids **(**Table 2**)**. Its primary geographical distribution is within the Mediterranean region, in its different parts: central [32], western [33–39] and eastern Mediterranean [40–42]; with scarcer records in Northeast Atlantic waters [18, 43, 44]. In the Mediterranean, few of the previously mentioned records provided morphometrical data and/or illustrations, and never a solid comparison with *C. chrysophryi *s*sensu stricto* justifying the signalment of this Atlantic species in Mediterranean waters. Curiously *C. chrysophryi* has never been reported on the type-host. It appears that there is an error in identifying the type-host (Louis Euzet, personal communication *in* Kouider El Ouahed-Amine [33]). However, future investigations on the type-host *S. aurata*, from the type locality, Northeast Atlantic waters are warranted to ascertain this theory.

Hence, previous records of *C.* cf*. chrysophryi* or of *C*. *chrysophryi* (mainly 1. *C*. cf. *chrysophrii* of Jovelin and Justine [28] ex *P. acarne*; 2. *C*. cf. *chrysophryi* of Bouguerche et al. [17] ex the same host off Algeria; and 3. that of *C. chrysophryi* of Lablack et al. [29] ex *P. bogaraveo* off Algeria) should be referred to as *Choricotyle* sp., a potentially new species pending formal descriptions. The question that arises, is whether these isolates correspond to a single species. Overall, the divergence between the previously mentioned isolates ranged between 1–3%. Generally, interspecific and intraspecific variations of partial *cox*1 sequences in polyopisthocotylans ranged from 0.2 to 5.6% [12]. The divergence between *Choricotyle* isolates from the different hosts is below the interspecific threshold generally agreed for Polyopisthocotyla [12]. Thus, we consider that this clade corresponds to a single species, that should be formally described and illustrated.

A particular record of “*C. chrysophryi*” worth mentioning is that of Llewellyn [18], ex *P. bogaraveo* from the Irish Atlantic Slope. This record provided detailed illustrations and morphometrical data. Most interestingly, Llewellyn [18] highlighted and illustrated the presence of a terminal lappet in his specimens, which is lacking in the diclidophorids described by Van Beneden and Hesse [10]. Hence, regardless of the host of the specimens described by Van Beneden and Hesse [10], the two Atlantic populations are clearly different. Thus, the diclidophorid of Llewellyn [18] from *P. bogaraveo* is not conspecific with *C. chrysophryi sensu* Van Beneden and Hesse [10] and we herein refer to *Choricotyle* ex *P. bogaraveo* from the Irish Atlantic Slope as *C. chrysophryi sensu* Llewellyn [18] pending further examination of these specimens.

A second species reported on Atlantic sparids is *C. pagelli* described as *D. pagelli* Gallien, 1937 from the gills of the blackspot seabream *P. bogaraveo* (junior synonym of *P. centrodontus* (Delaroche) [45]) also in Northeast Atlantic waters, around the British Isles [8] (see Fig. 3). The description was based on a single specimen, however, this species was found and reported again on its type-host *P. bogaraveo* in Portuguese waters of the Northeast Atlantic (i.e. close to the type locality British Isles) [30] confirming thus its validity. One might be tempted to consider that the record of “*C. chrysophryi*”, designated here as *C. chrysophrii sensu* Llewellyn [18] on *P. bogaraveo* in the Irish Atlantic Slope and the Irish Sea, NEA [18, 43] are highly likely *C. pagelli*. However, *C. chrysophrii sensu* Llewellyn [18] can be easily distinguished from *C. pagelli* by having a terminal lappet, by the presence of a ring organ at the level of the female genitalia, and especially by the organization of the haptor. *Choricotyle pagelli* is readily distinguished from *C. chrysophrii sensu* Llewellyn [18] in that the origins of its anterior peduncles are contiguous whereas the origins of the anterior peduncles of the latter species are separated by the width of the body [18].

*Choricotyle* sp. defined in this study (see Fig. 1) is likely to be *C. pagelli*, given that the *Choricotyle* sp. clade includes a sequence of *Choricotyle* from *P. bogaraveo*, which is the type-host of *C. pagelli*. However, since the localities are separated (Mediterranean *vs*. Atlantic) we refer to it as *Choricotyle* sp. until molecular data from the type locality are available.

Another species with doubtful validity is *C. marionis* St. Loup 1885, described on the blotched picarel *Spicara maena* (L.) (referred to as *Maena vulgaris* Valenciennes) from Mediterranean waters [46]. Sproston [7] considered that this species must remain doubtful until it can be redescribed and figured. Ayadi [47] reported *Choricotyle* sp. on the gills of *S. maena* and *S. smaris* (L.), that the author distinguished from available records of *Choricotyle* from *P. acarne*, from the common pandora *P. erythrinus*, the common two-banded seabream *Diplodus vulgaris* (Geoffroy Saint-Hilaire), the annular seabream *Diplodus annularis* (L.), the white seabream *Diplodus sargus* (L.), and from the Saddled seabream *Oblada melanurus* (L.) by several morphometrical features such as the size of the haptor, the length of haptoral peduncles, clamps size, body size, and oral suckers size. However, Ayadi [47] was apparently unaware of the description of *C. marionis* from the same host and also from Mediterranean waters nor about the fact that *Spicara* spp. are considered currently members of Sparidae (as they claim that belonging to another host family, Centracanthidae is another distinctive feature). Hence *Choricotyle* sp. of Ayadi [47] is, in fact, *C. marionisi* and we reinstate the latter as a valid species.

## Conclusion

The taxonomic status of *C. chrysophryi* has been complicated by host misidentifications, geographic variations, and historical inconsistencies. Our study suggests that *C. chrysophryi sensu stricto* was originally described from *Sparus aurata* in Atlantic waters, yet has only been reported on other hosts in the Mediterranean. Based on the presence of closely related, but distinct taxa on sparid hosts in both the Mediterranean and Northeast Atlantic, we propose that previous records of *C*. cf. *chrysophryi* may require reassignment to *Choricotyle* sp., which may represent a new species pending further formal description. Additionally, our analysis of *C*. *chrysophryi sensu* Llewellyn (1966) from *Pagellus bogaraveo* in the Irish Atlantic Slope reveals notable morphological differences from *C. chrysophryi sensu* *stricto*, suggesting it could be a distinct taxon. Similarly, our findings support the validity of *C. pagelli*, originally described from *P. bogaraveo*, while clarifying the status of *C. marionis*, which was previously considered dubious. The comparison with *Choricotyle* sp. from *Spicara maena* indicates that *C. marionis* should be reinstated as a valid species. These findings underscore the need for continued morphological and molecular analyses to address taxonomic uncertainties within the *Choricotyle* genus. However, the limitations of this study include the need for further sampling and more comprehensive molecular work to fully resolve species boundaries and confirm the taxonomic status of other taxa.

## Footnotes

1 The number of tests was estimated from the figure of Gallien [8].
